# “When you give birth you will not be without your mother” A mixed methods study of advice on breastfeeding for first-time mothers in rural coastal Kenya

**DOI:** 10.1186/s13006-016-0069-6

**Published:** 2016-04-26

**Authors:** Alison W. Talbert, Moses Ngari, Benjamin Tsofa, Lazarus Mramba, Edward Mumbo, James A. Berkley, Martha Mwangome

**Affiliations:** Centre for Geographic Medicine (Coast), Kenya Medical Research Institute/Wellcome Trust Research Programme, P.O. Box 230, Kilifi, 80108 Kenya; University of Florida, PO Box 110410, Gainesville, FL 32611-0410 USA; County Chief Nursing Officer, Kwale County, P.O Box 200, Kwale, 80403 Kenya; Centre for Tropical Medicine and Global Health, Nuffield Department of Medicine Research Building, University of Oxford, Old Road Campus, Roosevelt Drive, Oxford, OX3 7FZ UK

**Keywords:** Exclusive breastfeeding, First-time mothers, Advisers, Breastfeeding problems, Insufficient milk, Co-sleeping

## Abstract

**Background:**

Exclusive breastfeeding for the first 6 months of life is currently recommended by the World Health Organization, but mixed feeding earlier than this commonly occurs in rural coastal Kenya. Mothers may receive conflicting advice on breastfeeding from various sources including health workers, relatives and community members. We aimed to find out how first-time mothers learn to breastfeed, who advises them on infant feeding and what advice they obtain in case of any breastfeeding problems.

**Methods:**

To identify advisers, a questionnaire on socio-demographic status, place of delivery, household members, education and help received on breastfeeding, breastfeeding problems, name of advisers and their relationship to the mothers was administered to 50 new first-time mothers in Jaribuni, Kilifi (population approximately 18,000). Summary statistics were obtained using frequencies, medians and interquartile ranges (IQR). Focus group discussions (FGDs) were held amongst 4 groups of mothers who had answered questionnaires; 4 groups of their named advisers; and 1 group of community health workers in order to explore breastfeeding practices, problems and advice given. FGDs were analysed by thematic framework analysis.

**Results:**

First-time mothers were young (median age 18, IQR 17–21, range 14–26 years) and 42 % were single. Living in extended families was the norm and married women lived with their husband’s family. All had a female family member or neighbour helping with childcare in the perinatal period. The main advisers on breastfeeding were their mother or older female members of their husband’s family. Married first-time mothers felt obliged to follow their mother-in-law’s advice to maintain good relationships and show respect within the household. Breastfeeding problems were reported by 80 % of respondents. Nipple pain (56 %) was the most reported problem, then breast engorgement (48 %) and insufficient milk supply (38 %). Most problems were treated at home without consultation with health workers. Concerns were raised about co-sleeping, breastfeeding whilst lying down, and insufficient milk supply. Advisers would like more information on breastfeeding in order to help mothers.

**Conclusions:**

Interventions to increase knowledge of, and facilitate optimal breastfeeding practices in first-time mothers should include those family members who advise and assist with childcare around the time of delivery.

**Electronic supplementary material:**

The online version of this article (doi:10.1186/s13006-016-0069-6) contains supplementary material, which is available to authorized users.

## Background

In 2002 the World Health Organization and UNICEF endorsed the Global Strategy for Infant and Young Child Feeding, which recommended exclusive breastfeeding for the first six months of life, then continued breastfeeding alongside appropriate complementary feeding until age 24 months or more [1]. The importance of exclusive breastfeeding to the child includes protection against diarrhoea, respiratory infections and reduced risk of infant and child death [2–4]. The Kenya demographic and health survey of 2014 found that 99 % of infants under 6 months received breastmilk, but only 61 % were exclusively breastfed [5] The proportion of infants who were exclusively breastfed at age 0–1 month was 84.1 %, but only 42 % at age 4–5 months [5].

In Kenya, mothers receive health advice during contacts with health workers at clinics and antenatal and maternity services, and also from their families and communities, whose social norms around infant feeding may be incompatible with current recommendations [6, 7]. Living in extended families has been reported to be associated with reduced duration of exclusive breastfeeding [8]. In sub-Saharan Africa, mothers receive advice on nutrition and childcare from older female family members who are considered experts as a result of having raised their own children [9, 10]. Husbands may expect their wives to follow the advice given by these older women whose influence within the household may undermine young mothers’ infant feeding decisions that seek to follow health professionals’ advice [10, 11]. A study of roles and responsibilities in newborn care in 4 sites in Nigeria, Tanzania and Ethiopia further reported that older women considered first-time mothers to be in need of training in childcare, and these mothers took longer to assume full care of their babies after birth than multiparous women [12].

Research in developed countries suggests that a large proportion of mothers experience concerns in establishing breastfeeding in the early neonatal period and they may consult multiple sources of advice [13, 14]. There have been fewer studies on how first-time mothers in developing countries try to resolve their breastfeeding problems [7, 15]. We aimed to identify the advisers on breastfeeding to first-time mothers, understand how they become advisers and what type of advice they give.

## Methods

### Study site

The study was located in the former administrative unit of Jaribuni Division, population of approximately 18,000, in Kilifi County, Kenya. The population is mainly subsistence farmers in a semi-arid zone with food insecurity. Marriages are patrilocal, that is, usually the bride moves to live with her husband’s family. Apart from roadside trading areas, most houses are grouped in small clusters of less than 10 houses of an extended family. There is one government dispensary with 2 professional nurse-midwives and the nearest hospital is the Kilifi County Hospital (KCH), 30 kilometres away on a rough, poorly-linked road with infrequent public transport. The journey from Jaribuni to KCH takes approximately 2 h. In Kilifi North Subcounty in 2014, 66 % of skilled deliveries took place in Kilifi County Hospital and 34 % in rural health facilities (personal communication from sub-county records office) but there were no data available on the number of home births at sub-county level. The study site was chosen after consultation with the local District Health Management Team on account of its low utilization of health facility services, high rates of malnutrition and poor transport links with the district (now county) hospital.

### Ethical clearance

Ethical clearance for the study was obtained from the Kenyan Medical Research Institute Ethical Review Committee in Nairobi.

### Study procedures

**Fig. 1:**
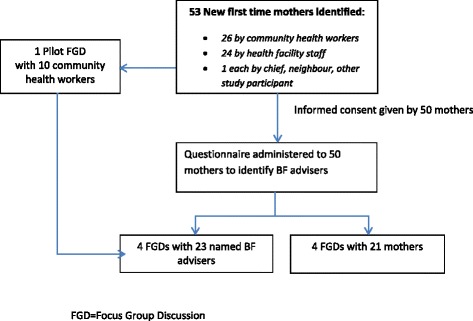
Flow diagram of study of activities

See Fig. 1 for flow diagram of study activities.

### Recruitment of first-time mothers

Using demographic surveillance data [16], it was estimated that approximately 90 women living in Jaribuni Division would become first-time mothers within a year. The study aimed to recruit a minimum of 50 first-time mothers. Community sensitization meetings were held prior to the study. Local administration leaders, dispensary staff and community health workers (CHWs) were asked to notify the study team of first-time mothers with newborn babies. Identified mothers were first visited by the CHW, who explained the aims of the study and for those mothers who agreed to participate, a date was booked for a second visit. At the second visit, mothers were seen at home by the CHW and the principal investigator. A written consent was obtained by the CHW in the mother’s preferred language. A questionnaire was administered in the mother’s preferred language by the CHW. To avoid bias, mothers were encouraged to answer the questionnaire without consulting other household members. Home visits were carried out between June 2013 and September 2014.

### The questionnaire

The questionnaire aimed at identifying the key advisers on infant feeding for first-time mothers. The questionnaire collected information on demographic characteristics; current infant feeding practices, including breastfeeding initiation and challenges; and key advisers: see Additional file 1. The questionnaire was developed, translated into the local language and piloted with mothers on the postnatal ward at KCH. During administration, specific problems on breastfeeding were probed. Household members were listed with name and relationship of occupants, together with details of those who had helped with care of the newborn and who advised on breastfeeding.

### Focus group discussions

Information from the questionnaire responses was used to identify and invite different groups of mothers for FGDs. After preliminary analysis, different groups of first-time mothers emerged; young, single and married mothers, those who had given birth at home and in health facilities, and those who had practiced exclusive breastfeeding and those who had not. Additionally, questionnaire-identified home-based advisers for the first-time mothers were separately invited for discussions. Professional health worker advisers were not included in the discussion groups because only a small number of trained staff was named in response to the questionnaire with a widespread geographical distribution.

In order to explore further the choice of advisers and the type of advice given, first-time mothers and named advisers were invited to participate in FGDs. A discussion guide was developed and the topics were first piloted with a group of CHWs who had assisted with the questionnaire administration: see Additional files 2 and 3. Topics included practices after childbirth, help to initiate breastfeeding, advice and treatment given for feeding problems, benefits of and challenges to exclusive breastfeeding, when milk is “bad” for the baby, insufficient milk supply, decision making around introduction of food and fluids to the baby, changes in advice on infant feeding since the time the advisers had their babies. We also discussed childcare and infant feeding options when the mother returns to school or work. The discussions were held in November 2014 and February 2015 in local churches with 4 groups of the first-time mothers and 4 groups of their advisers.

Individual written informed consent was obtained after reading the study information sheet at the start of each FGD. An independent witness was nominated by the illiterate advisers to verify the procedure and sign on their behalf. The discussions held in the local language, Kauma, were moderated and recorded digitally by an experienced research assistant. Notes were taken by another assistant who transcribed and translated the transcripts into English, then checked by one of the investigators.

### Data analysis

Questionnaire results were coded and entered on an Epidata v1.4.2 database then transferred to Stata 12 for analysis. Summary statistics were calculated using counts, proportions, medians and interquartile ranges. Thematic analysis of the focus group discussion data was carried out using the framework method [17]. The English transcripts were read and independently coded by two of the authors who then met to develop an analytical framework. The source data were coded using NVivo 10 software and categories of related codes were summarized using framework matrices in order to examine the similarities and differences within and between the discussion groups. Overarching themes were sought using a combination of deductive and inductive analysis.

## Results

In total 53 first-time mothers were identified; 26 by the CHWs, 24 by the health facility staff, 2 by other study participants one of whom was also a neighbour and 1 through the chief. Fifty mothers consented to participate in the study. Amongst the three women who did not participate, one reported that her husband had refused permission and another said she was waiting for her husband to return before deciding.

### Questionnaire results

The respondents’ ages ranged from 14 to 26 years with a median of 18 years (interquartile range 17–21). Twenty nine mothers (58 %) were married. All except one had been to school, but 25 (50 %) had not completed 8 years of primary level education. Religious affiliations were Christian 24 (48 %), none 15 (30 %) and Muslim 11 (22 %).

Most women (45/50 90 %) were visited in their own home, the remainder were interviewed in other homesteads where they were staying temporarily. Of the 21 single mothers, 19 (90 %) were living with their mother and 2 (10 %) with aunts. Of the 29 married women, 20 (69 %) lived with their mother-in-law in their husband’s family homestead. The majority of the husbands were working elsewhere and visiting at weekends. The husbands were present all week for 14 (48 %) respondents. Only 2 of the married women lived with their husband and child without other family members.

Of the 50 mothers, 42 (84 %) had delivered in a health facility: (20 (40 %) in hospital, 3 (6 %) in a health centre and 19 (38 %) in a dispensary) and 8 (16 %) home births were reported. Of the hospital births, 17 (34 %) took place in Kilifi County Hospital, 1 (2 %) in a church hospital in the next district and 2 (4 %) in the regional referral hospital in Mombasa The home births were conducted by an aunt in 3 cases; traditional birth attendant 2; mother, mother-in-law and maternal grandmother 1 each. Five infants were born with a weight less than 2.5 kilograms.

Only 13 (26 %) mothers reported having been given their infant to hold immediately after the birth. The reported median time to first holding their babies was 0.5 hours (interquartile range 0.17 to 2 h). Mothers delivering at the local dispensary reported that their child was taken away by a nurse to be weighed first. Twenty five (50 %) mothers successfully fed their baby within 1 h (interquartile range 0.5 to 3 h) and 46 (92 %) within 24 h of birth.

The majority of the single mothers (19/21 90 %) reported that assistance with care for the infant was given by their mothers, one named her aunt and another was helped by a neighbour. Amongst the married mothers, four (14 %) received help from both their mother and mother-in-law whilst eleven (38 %) each received help from mother or mother-in-law only.

At the time of participation in the study the median age of the infant was 22 days (interquartile range 16–42) and the age range was from 4 to 106 days.

### Infant feeding practices

When asked if colostrum was good for the baby’s health 28 (56 %) mothers replied “Yes”, 4 (8 %) “No” and 18 (36 %) did not know. Other fluids were given before breast milk to 8 (16 %) babies; 2 of these following home births but also in 6 babies born in health facilities (5 in a dispensary and 1 in a health centre). These fluids were predominantly sugar water but one baby received coconut water and another tea.

At the time of the questionnaire, all but one child were breastfeeding (98 %), though only 31 (62 %) reported exclusive breastfeeding. Three babies were receiving cows’ milk: two mothers were supplementing because they felt their milk was insufficient and one mother had been advised to stop breastfeeding so she could leave the baby with relatives and return to school. No babies had received formula milk. Other fluids given were water, sugar water and a solution of sugar and salt. Two babies who were seen at 67 and 106 days of age had started on maize porridge.

Mothers were asked if they had received any practical help with breastfeeding technique and 23 (46 %) reported that they had. Professional midwives assisted 11 (22 %) mothers; ten respondents were helped by their mothers, one by her mother-in-law and one by an aunt.

Only eleven (22 %) mothers reported that they had received teaching on breastfeeding at antenatal clinic. Seven (14 %) women had been taught before the birth by their mother, 5 (10 %) by their mother-in-law and 15 (30 %) said they had learned by observing other women. Only one (2 %) mother had learnt about breastfeeding in school and one from the media.

### Breastfeeding problems

Forty (80 %) of the mothers reported at least one problem in breastfeeding (Table 1).

**Table 1:** Breastfeeding problems as reported by mothers on questionnaires

Breastfeeding problem*	Number of women (%)	Had sought advice (%)
Knowing what to give for first feed	3 (6)	1 (2)
Getting the baby to suck	8 (16)	8 (16)
Insufficient milk	19 (38)	10 (20)
Nipple pain	28 (56)	13 (26)
Breast engorgement	24 (48)	11 (22)
Breast abscess	1 (2)	1 (2)

*Mothers could report more than 1 problem

*N* = 50

### Who advises first-time mothers?

Twenty two mothers (44 %) reported that they had asked for advice for a breastfeeding problem. Eight of ten single mothers sought advice from their mother, one from a community health worker and one from a professional health worker. Of the married mothers, four sought advice from a health worker, two from their sister-in-law (husband’s brother’s wife), two from their mother-in-law, two from their mother, one from both mother and mother-in-law and one from her mother, aunt and father-in-law’s brother’s wife.

### Focus group discussion participant characteristics

The community health workers’ group consisted of 8 female and 2 male members all of whom had at least some primary education as did the 21 mothers but it was notable that only 4 out of 23 advisers had any formal education, including adult education. The age of the CHWs ranged from 27 to 46 years (median 40) and the mothers 15 to 29 years (median 19), the age of the advisers, all female, ranged from 35 to 55 years but 14 advisers did not know their age.

### Focus group discussion findings

From the discussions, three main themes emerged as discussed below.

### Balancing family relationships

There exists a complex relationship between the first-time mothers and their advisers. Because the majority of the key advisers are relatives, first-time mothers felt that it was important to maintain good relationships within the household.

“*You have to listen to whatever your mother in law and your husband will tell you, you should get on well with the people you live with*.” Mother FGD2

Newly married first-time mothers have moved into the compound of their husband’s family and feel at risk of being sent away if they are perceived to be disobedient or are not taking good care of the baby.

*“I was forced to decide that he should be given some water because she will say that I am making her grandchild suffer and I will have to go back to our home, so I said it is OK for my baby to be given some water.”* Mother FGD2

The married women were asked how they would choose between their mother and mother-in-law’s advice. Most answered that they were expected to listen to their mother-in-law because she lived with them, was very supportive from the time of delivery, and helped them with childcare and domestic work whereas their mother was only a visitor.

*“She is the one we are together with.”*

“*She is the one who performs all the duties for me that’s why I’ll follow her advice.*” Mothers FGD3

Other mothers were able to negotiate with those offering home advice which differed from that of the hospital staff, citing possible risks to the baby and fear of criticism from health workers.

*“I’ll tell her no, do you know what they have seen at the hospital that they told me this? We’ll quarrel a bit. We’ll disagree, because the doctor has told me not to do such things, because he saw there might be danger ahead. I will not agree with what my mother is telling me. Now in case you gave him, then a problem arose, you went to the doctors and you were asked where the problem came from and you said what you did, you as the mother you’ll be accused, so you were supposed to oppose the home advice”* Mother (unmarried) FGD1

Mothers experienced pressure from others to introduce porridge before 6 months of age if their babies cried a lot, particularly at night. Despite most of the advisers and mothers knowing that exclusive breastfeeding is recommended for the first 6 months of life, mothers were criticized by family members and neighbours for not introducing other foods earlier. Advisers in several groups reported that it used to be the norm to start the baby on porridge even in the first month of life. Mothers following hospital advice to breastfeed exclusively felt that this practice might be perceived by others as harmful to the baby.

*“The baby was crying; that’s when the neighbours told me to give him food because the milk wasn’t enough.”* Mother FGD3

*“It is bad on the other side, when he is taken by other people you are always alert, you want to see what is done to him, so people may not understand you. Sometimes people talk a lot, like you are starving the baby, he has grown and you are just breastfeeding, he is crying because of hunger, so you are blamed. First it is very hard breastfeeding exclusively for 6 months; you’ll see the six months are very long because people are against whatever you are doing, so you have to be firm. It is bad but not for the child, but on our side as parents.”*

*“You are competing with the people who gave birth before you and this is your first pregnancy.”*

“*You’ll hear many things and you have many challenges.*” Mothers FGD4

First-time mothers reported that their lack of experience made it hard to challenge home advice.

*“Because it’s your first-time then you have to be tough to get your way, not to follow whatever you are told by your family. We have more challenges because it’s something we don’t know. Our parents have gone through it and they are the ones who are telling you to give him sugar water, and you say you’ll not give him, you have to be tough.”* Mothers FGD4

### Learning under close supervision

The first few days after the baby is born the new mother is in a privileged position; she is allowed to rest from her normal duties and someone cooks for her and washes her and the baby’s clothes.

From the questionnaire results and discussion groups’ findings, it was apparent that each mother had a female helper in attendance around the time of birth and during the early postnatal period. The mothers described the helper’s role was to teach by demonstration the skills needed to hold and breastfeed the child and there was consensus that learning took place by practice under close supervision by the helper. A few of the mothers who had given birth in a health facility also mentioned that they were shown by the midwife how to breastfeed soon after the birth.

*“When you give birth you will not be without your mother, your grandmother, any parent.”* Adviser FGD2

*“Nobody advised me, it’s only my mother who was close to me; she explained it to me in case anything wasn’t clear.”* Mother FGD1

The advisers described that their role after home births was to bathe the mother and baby and look after the baby in order for the mother to rest after delivery. They explained that the time to first feed depended on how settled the baby was, the cue to breastfeed was when the baby became restless or started to cry. Generally it was thought that the baby needed help to attach to the breast. The only benefits of skin-to-skin contact elicited were that the baby could learn its mother’s smell and keep warm.

*“If the child tries to find it (the breast) himself, he’ll get frustrated and cry, how will he look for it, and the child doesn’t know?”* Adviser FGD1

The helpers would sometimes advise the mother to give other fluids in the first days of life if they were concerned that the milk was not enough.

*“They were doing it for two reasons: one, the colostrum will upset the baby’s stomach, two if there is no milk coming out what will the baby drink and the baby should get sugar water while waiting for the milk to come in.”* CHW FGD

Helpers would sometimes take over the care of the baby to the extent of wet nursing and sleeping with the baby. Wet nursing is reportedly less common than previously because most people are aware of the risk of HIV transmission.

*“There are other babies like my grandchild who cried a lot after delivery and he didn’t want to suck from his mother so I had to breastfeed and sleep with him.”* Adviser FGD2

*“I used to breastfeed my grandchildren, I breastfed them for a long time but now you cannot breastfeed anyone else’s child.”* Adviser FGD4

### Breastfeeding challenges

The advisers were asked about their role in management of breast problems during the first few weeks of breastfeeding. Nipple pain, mastitis, cracked nipples were discussed; the advisers usually recommended the mother to persevere with breastfeeding. Home remedies included buying paracetamol from the shop and expressing milk to reduce engorgement. Mastitis led to some mothers discarding the milk and only feeding from the unaffected side. Other mothers were referred to the local dispensary for treatment of sores or abscess.

*“My daughter, after giving birth, she got sores on both breasts when the baby was breastfeeding and I told her to go to the dispensary.”* Adviser FGD4

Seven of the nine groups mentioned the risk of suffocation of the baby from poor breastfeeding technique and bed-sharing. The advisers said it was important for the mother to know how to attach her baby to the breast so that it would not squash the baby’s nose. The mother should also look at her baby whilst feeding and not get distracted otherwise the milk might flow too fast for the baby to swallow. The first few days after the birth, she should sit up to breastfeed in case she fell asleep.

*“At night you should not breastfeed when you are asleep.”*

“*Maybe you can fall asleep and the milk can flow too much.”*

“*The milk can flow too much and get into the baby’s nose.*” Mothers FGD3

Advisers taught mothers how to position their baby when sleeping with them. They said there was a risk of babies dying from tired mothers overlaying them and from milk aspiration if mothers fed whilst asleep. Some advisers mentioned that they slept with the baby and took the baby to the mother for feeding when he started to cry.

*“If she gave birth you have to teach her, the first thing is breastfeeding, she gets hold of the baby and breastfeeds. After breastfeeding when its time for sleeping, because she is not used to sleeping with the baby you will sleep with him, then you will teach her, If you are sleeping you sleep with him like this, you keep the baby up a little and you down so that your breasts will not cover him.”* Adviser FGD3

## Discussion

This study found that the main source of advice for breastfeeding problems in first-time mothers was other female family members at home, rather than trained health workers in the dispensary or hospital. Each new mother had a helper who stayed with her around the time of the birth and taught her how to hold the baby and breastfeed. She was the one to advise on problems as they arose. Who became the main adviser depended on which older woman with experience of raising children was chosen to help the new mother after the baby was born, was staying with her and was around to offer ongoing advice alongside practical supervision. Most minor breastfeeding problems were managed at home with advice to the mother to persevere.

Antenatal education on breastfeeding was limited and there were no routine home visits to new mothers by professional or community health workers. Rather than a few health workers or expert community members being recognized and consulted widely on infant feeding, it seemed that each first-time mother drew on the experience of her home-based helper in the days after the birth for support and advice on baby care and breastfeeding in the first instance.

There was little understanding of the rationale of skin-to-skin contact for initiation of breastfeeding and there were anecdotal reports that mothers had been discharged from health facilities before the first feed. Changes in delivery room routines are required so that the mother is given the baby to hold immediately after birth and midwives explain to the mother about the baby’s rooting reflex, ability to latch on and the benefits to mother and baby of early breastfeeding [18–20]. The high proportion of mothers experiencing nipple pain and engorgement implies the need for more assessment of latching and observation of feeds by trained staff to prevent sub-optimal breastfeeding skills.

There was pressure on first-time mothers at home to give extra fluids, including porridge for perceived hunger or thirst before the recommended age. As found in other studies in Kenya and South Africa, excessive crying was taken as a sign that the mother’s milk supply was insufficient [6, 21]. Increasing the frequency of feeds was not used as a strategy for increasing milk supply outside the first week of life. There is a need to improve knowledge of mothers and their advisers of the demand–supply feedback loop of breast milk production and to empower mothers to be confident in their own body’s capacity to meet the child’s nutritional requirements [22].

The first-time mothers in this rural community were able to rely on the presence of older female family members to assist with childcare but this also led to fears that these older women might directly give fluids or food against the wishes of the new mother. It seemed that some advisers kept the baby with them and only gave it to the mother to breastfeed when they perceived the child to be hungry. Whilst the intention was to allow the mother to rest this could lead to less frequent breastfeeding than if the mother was with the baby all the time. This in turn leads to reduced breast stimulation and less milk production.

Safety issues around co-sleeping and aspiration of milk from unregulated milk flow during feeds were mentioned by most groups. Mothers and advisers were concerned that newborn babies might aspirate milk if the mother did not pay attention whilst breastfeeding. This was also cited as a risk if a tired mother fell asleep whilst breastfeeding so lying down to feed was discouraged. To our knowledge this has not been highlighted as a major concern in other studies in developing countries and requires further in-depth exploration. Accidental asphyxia has been reported as a rare outcome of mothers falling asleep whilst breastfeeding in bed [23]. There has been recent debate on the hazards of bedsharing following American recommendations on safe sleeping environments to reduce sudden infant deaths [24, 25]. The advisers’ descriptions of sleeping with the newborn babies, together with the frequent warning of the risks to the baby of rapid milk flow and breastfeeding whilst lying down suggest that babies may not be receiving as many night feeds as if they were sleeping with their mothers [26].

Factors supporting breastfeeding in our study population are that mothers are encouraged to stay at home to look after their babies, and the limited availability and uptake of outside employment. The expectation that they will stay at home contrasts with urban mothers in informal settlements in Nairobi, who go out to work leaving their babies in childcare facilities from an early age [27].

In this rural community most respondents were living in extended families of three or more generations and very few mothers lived in nuclear families with their husbands. Intergenerational hierarchies led to adoption of grandmothers’ advice out of respect. Some of the advisers admitted they were at a loss to know what to feed the babies and expressed a desire to update their knowledge now that wet nursing and early mixed feeding was discouraged. There is a potential role for community health workers, who are often older women, in information sharing with advisers at home on current feeding recommendations, with the proviso that adequate time is taken for explanation and dialogue rather than relaying instructions. Grandmothers included in nutrition education projects elsewhere have been found to incorporate new nutrition knowledge into their practices and positively influence mothers to change infant feeding behaviour [11].

Our study findings emphasize the important role of advisers in the home in supporting or contradicting health workers’ advice on exclusive breastfeeding to provide optimum nutrition for the first 6 months. Young mothers need well informed, sympathetic advice and encouragement to continue with frequent breastfeeding in the face of anxieties about milk supply. Home based follow up visits by community health workers trained in nutritional counselling and peer support groups for mothers are two promising approaches for improving mothers’ breastfeeding self-efficacy and confidence [28, 29].

The limitations of our study are the small number of mothers and limited geographical area of the study, which was chosen for its difficulties accessing health services. We chose not to invite the professional health workers to contribute to focus group discussions partly because of logistical reasons but also because we were primarily interested in mothers’ advice from relatives and community members who were not trained health workers. We were unable to trace some of the mothers for the focus group discussions due to the lag time from the questionnaire, and some were reported to have moved out of the study area following marriage and others resumed schooling, hence these groups might be under-represented in the discussions. Despite these limitations we feel that our recommendations as summarized in Table 2 could be applied to first-time mothers in other rural areas where extended families living together, low female education and employment levels and early marriage are prevalent.

**Table 2:** Summary of recommendations to support early and exclusive breastfeeding

*1.*	*Improve antenatal teaching on breastfeeding to pregnant women and include other family members who will attend them in the perinatal period*
*2.*	*Introduce the concept of skin-to-skin contact after birth to pregnant women and health workers and alter postnatal care routines to allow babies time to initiate early breastfeeding*
*3.*	*Provide training for health workers to support exclusive breastfeeding to mothers on postnatal wards and for health workers and community health workers to counsel mothers and support exclusive breastfeeding once the mother returns home*
*4.*	*Explain physiological principles of on-demand feeding and stimulation of milk production to mothers and other family members attending them*
*5.*	*Explain how to recognise the signs of adequate/inadequate milk supply to mothers and other family members attending them*

Our study has highlighted the breastfeeding problems encountered by a small group of first-time mothers, that would not have been reported to nurses or community health workers, and their use of home-based supporters. Larger scale mixed methods studies could measure the burden of breastfeeding problems in first-time African mothers and enable a better understanding of the issues around learning to breastfeed. There is a need for further research to identify modifiable barriers to early and exclusive breastfeeding in order to implement contextually appropriate interventions.

## Conclusion

Senior female family members were essential companions of first-time mothers around the time of birth and in the early post-natal period providing practical assistance to mother and baby. Their influence as teachers and advisers on breastfeeding should be acknowledged so that they are included in initiatives to improve knowledge on and strengthen practices of early and exclusive breastfeeding in the first 6 months of life.

## Additional files

Additional file 1: Questionnaire for mothers. (DOCX 19 kb) Additional file 2: Topic guide for focus group discussions with mothers. (DOCX 14 kb) Additional file 3: Topic guide for focus group discussions with advisers. (DOCX 15 kb)
