# Supplementary material for: “When you give birth you will not be without your mother” A mixed methods study of advice on breastfeeding for first-time mothers in rural coastal Kenya
Source: Int Breastfeed J. 2016 Apr 26;11:10. doi: 10.1186/s13006-016-0069-6 (PMC4845378; doi:10.1186/s13006-016-0069-6)
Supplement: Additional file 1: — Questionnaire for mothers. (DOCX 19 kb) [file 13006_2016_69_MOESM1_ESM.docx]

**English questionnaire for mothers with newborn babies**

|  | **Initials** **of respondent _ _ _ Study number _ _ _** |
| --- | --- |
|  | **Age Education level Marital status Religion** |
|  | **Date of questionnaire (dd/mmm/yy)** __/__/___ **Initials of fieldworker _ _ _** |
|  | **Birth** |
|  | When did you give birth? Date­__/__/___ Time __(hr)__ (min) (*24 hour clock)*  Place of birth:  ( *home or hospital/ other type of health facility- give name)*  Birthweight _ .__kg *from healthcard*  Tick if not known or weighed >48 hours after birth  Premature or LBW- *tick box if either applies* |
|  | What number baby is this? |
|  | Who assisted or attended to you during delivery? (*tick as appropriate) Write their name*  Mother  Mother-in-law  Other family member (*specify relationship)*  Neighbour  Traditional birth attendant  Community health worker  Trained health worker ( health facility births)  Other (*specify relationship)* |
|  | How long after birth did you first hold your baby?  Hours _ _ or mins_ _( if less than 1 hour) |
|  | **Early breastfeeding** |
|  | How long after the birth did the baby first feed?  Hours _ _ or mins_ _( if less than 1 hour)  Did you give any fluids before starting to breastfeed?  Yes  No  Are you currently giving any fluids (water, glucose etc other than breast feeding)  Yes What kind of fluid? _ _ _ _ _ _ _ _ _ _ _ _ _ _ _ _ _  No |
| **6.** | Have you had any practical assistance from anyone on how to breastfeed?  Yes  No  If yes, who was it? *Write their name as well as indicating relationship*  Mother  Mother in law  Aunt  Sister  Neighbour  Friend  Other *( name and* *describe relationship to respondent*) _ _ _ _ _ _ _ _ _  Whose assistance was most useful? ­_ _ _ _ _ _ _ _ _ _ _ _ _ _ _ _ |
|  | **Knowledge of and influences on breastfeeding** |
| **7.** | Did you receive any teaching on breastfeeding before the baby’s birth?  Yes  No  How did you learn about breastfeeding before the birth? (*tick those applicable)*  Health education at antenatal clinic  Given advice by relative *( name and* *describe relationship to respondent*) _ _ _ _ _ _ _ _ _  Given advice by friend (*name*) _ _ _ _ _ _ _ _ _  Given advice by other person *(name)*_ _ _ _ _ _ _ _ _  Taught at school  From the media *(radio/TV/newspapers/magazines)*  Observing other mothers breastfeeding  Other method *(describe)* _ _ _ _ _ _ _ _ _  Do you think the first milk (colostrum) is good or bad for the health of the baby?  Good  Bad  Don’t know |
|  | **Household support** |
| **8.** | Is this your normal residence?  If no, whose is it? ­_ _ _ _ _ _ _ _ _ _ _ _ _ _ _ _ _ _ _ _ _ _ _ _ _ _ _ _ _ _  Whose house did you stay in from the birth of the baby until now?  Who is staying there now? *(list starting with head of household)*   \|  \| Name \| Relationship \| \| --- \| --- \| --- \| \| 1 \|  \|  \| \| 2 \|  \|  \| \| 3 \|  \|  \| \| 4 \|  \|  \| \| 5 \|  \|  \| \| 6 \|  \|  \| \| 7 \|  \|  \| \| 8 \|  \|  \| \| 9 \|  \|  \| \| 10 \|  \|  \|   If not your usual residence, who do you normally live with?   \|  \| Name \| Relationship \| \| --- \| --- \| --- \| \| 1 \|  \|  \| \| 2 \|  \|  \| \| 3 \|  \|  \| \| 4 \|  \|  \| \| 5 \|  \|  \| \| 6 \|  \|  \| \| 7 \|  \|  \| \| 8 \|  \|  \| \| 9 \|  \|  \| \| 10 \|  \|  \| |
| **9.** | Who has been helping you look after the baby since the birth?_ _ _ _ _ _ _ _ _ *Write their name*  Mother  Mother in law  Aunt  Sister  Neighbour  Friend  Housegirl  Other (*describe*) |
| **10.** | How much longer will they help you?  Weeks _ _ or days _ ( *if less than 1 week* ) Other _ _ _ _ _ _ _ _ _ _ _ _ _ |
|  | **Problems** |
| **11.** | Have you had any problems or concerns about the health of your baby?  Yes  No  If yes, what were they? _ _ _ _ _ _ _ _ _ _ _ _ _ _ _ _ _ _ _ _ _ _ _ _ _ _ *(Probe )*­  Did you have a problem:  Knowing when to start breastfeeding?  Yes  No  Knowing what to give for the first feed?  Yes  No  Getting the baby to suck?  Yes  No  Not having enough milk?  Yes  No  Pain in nipples on breastfeeding?  Yes  No  Pain from breast engorgement?  Yes  No  Breast abscess?  Yes  No |
| **12.** | If yes, who did you go to for advice? *(Tick as appropriate & write their name*)  Mother  Mother in law  Aunt  Sister  Neighbour  Friend  Health care worker  Local midwife ( traditional birth attendant)  Other *( name and* *describe relationship to respondent*) _ _ _ _ _ _ _ _ _  Whose advice did you follow? *(Tick as appropriate & write their name*)  Mother  Mother in law  Aunt  Sister  Neighbour  Friend  Health care worker  Local midwife ( traditional birth attendant)  Other ( *name and describe relationship to respondent*) |
| **13.** | Are there any other important issues concerning looking after a new baby that you think we have forgotten to ask? *(Probe)* |
