# Supplementary material for: “When you give birth you will not be without your mother” A mixed methods study of advice on breastfeeding for first-time mothers in rural coastal Kenya
Source: Int Breastfeed J. 2016 Apr 26;11:10. doi: 10.1186/s13006-016-0069-6 (PMC4845378; doi:10.1186/s13006-016-0069-6)
Supplement: Additional file 2: — Topic guide for focus group discussions with mothers. (DOCX 14 kb) [file 13006_2016_69_MOESM2_ESM.docx]

**Topic guide for focus group discussions with new mothers**

**Introduction**

In this research we want to explore the advice available to inexperienced mothers on how to feed their babies. We plan to do this by identifying advisers of new mothers using the social network analysis approach then hold group discussions with these advisers. We would also like to hold group discussions with the mothers themselves to learn how they ask for and receive advice and how they decide which advice to follow.

**Suggested topics (in bold), questions (in normal type) and probes (in italics) to include:**

**Attitude to breastfeeding and influences before the birth**

What is the role of breastfeeding in the health of the baby and mother?

What are the advantages and disadvantages of exclusive breastfeeding?

**Preparedness for looking after a newborn baby**

Did mother feel confident before the birth about looking after her baby?

**Difficulties encountered in looking after newborns**

Any aspects of caring for baby when mother felt unsure what to do?

Any problems in establishing breastfeeding?

*Knowledge of how to get baby to attach.*

*Concerns about quality/quantity of milk.*

Did mother have any problem with engorged breasts or painful nipples?

*What remedies were advised, by whom?*

Was the baby given fluids or medicines other than breastmilk?

*Why?*

**Sources of advice for new mothers**

Who do they ask for advice?

*Ask them to give relationship. Do they live close or far away? Ask in person or by phone?*

Who gives unsolicited advice?

*What kind of advice is offered?*

If the mother does not want to follow the advice how do they handle the situation?

*How do they decide whose advice to follow?*
