# Supplementary material for: “When you give birth you will not be without your mother” A mixed methods study of advice on breastfeeding for first-time mothers in rural coastal Kenya
Source: Int Breastfeed J. 2016 Apr 26;11:10. doi: 10.1186/s13006-016-0069-6 (PMC4845378; doi:10.1186/s13006-016-0069-6)
Supplement: Additional file 3: — Topic guide for focus group discussions with advisers. (DOCX 15 kb) [file 13006_2016_69_MOESM3_ESM.docx]

**Topic guide for focus group discussions with advisers of new mothers**

**Introduction**

In this research we want to explore the advice available to inexperienced mothers on how to feed their babies. We plan to do this by identifying advisers of new mothers using the social network analysis approach then hold group discussions with these advisers. The aim is to find out knowledge, attitudes and practices of advisers in relation to initiating breastfeeding and what they advise in case of any problems with feeding young infants.

**Suggested topics (in bold), questions (in normal type) and probes (in italics) to include:**

**Common and accepted practices immediately after birth**

Practices immediately after the birth.

*Rationale of identified key practices related to feeding, hygiene, contact with mother.*

**Starting to breastfeed**

When to start breastfeeding.

*Rationale for when to start breastfeeding.*

What help does a first time mother need to breastfeed?

*What are the common problems?*

*How do advisers handle the situation: explanation/demonstration?*

**Breastmilk**

Opinions on giving colostrum: benefit/risks.

*Effects of colostrum on the baby.*

*What happens to the colostrum- is it discarded?*

Situations when mother’s milk may be bad/harmful for the baby.

*Infections or behaviour of mother.*

Mother does not have enough milk.

*Signs and solutions to the problem.*

Any special diet a mother needs while breastfeeding/ strategies to increase milk production?

*Special foods, quantities, frequencies.*

*How to measure success of strategy.*

**Giving other fluids to baby**

Are any other fluids necessary for the baby or recommended in particular situations.

**Infant feeding practices**

When to start introducing other foods to infant.

What foods do you recommend/not recommend for the first year of life?

*Reasons for giving/not giving certain foods.*

**Changing advice over time**

Has advice on breastfeeding and infant feeding changed since you were a new mother?

*If it has, what do they think of the new advice?*

*Sources of new information: other local women, clinic, NGOs, media etc.*

What would make you change the advice you give?
